# Association of Embolic Sources With Cause-Specific Functional Outcomes Among Adults With Cryptogenic Stroke

**DOI:** 10.1001/jamanetworkopen.2018.2953

**Published:** 2018-09-28

**Authors:** Fumi Kiyuna, Noriko Sato, Ryu Matsuo, Masahiro Kamouchi, Jun Hata, Yoshinobu Wakisaka, Junya Kuroda, Tetsuro Ago, Takanari Kitazono

**Affiliations:** 1 Department of Medicine and Clinical Science, Graduate School of Medical Sciences, Kyushu University, Fukuoka, Japan; 2 Department of Health Care Administration and Management, Graduate School of Medical Sciences, Kyushu University, Fukuoka, Japan; 3 Center for Cohort Studies, Graduate School of Medical Sciences, Kyushu University, Fukuoka, Japan; 4 Department of Epidemiology and Public Health, Graduate School of Medical Sciences, Kyushu University, Fukuoka, Japan; 5 Cerebrovascular Division, Cerebrovascular and Neurology Center, National Hospital Organization Fukuoka-Higashi Medical Center, Koga, Japan

## Abstract

**Importance:**

It is unknown whether poststroke outcome varies between different potential causes in patients with cryptogenic stroke.

**Objective:**

To investigate whether functional outcome differs according to potential embolic sources after cryptogenic stroke.

**Design, Setting, and Participants:**

This multicenter, hospital-based, prospective stroke registry cohort study investigated potential embolic sources on admission and assessed 3-month outcome in patients with ischemic stroke hospitalized at 7 stroke centers in the Fukuoka Stroke Registry. This registry enlisted 9866 consecutive patients with acute ischemic stroke who were enrolled from June 11, 2007, to May 31, 2016, in Fukuoka, Japan. Patients with small vessel occlusion (n = 3130), extracranial and intracranial atherosclerosis causing at least 50% luminal stenosis in arteries supplying the area of ischemia (n = 2011), and other specific uncommon causes of stroke identified (n = 301) were excluded. Potential embolic sources were diagnosed in patients with embolic stroke of undetermined source (ESUS) based on the following criteria proposed by the Cryptogenic Stroke/ESUS International Working Group: minor-risk potential cardioembolic sources (MCS) (n = 209), covert paroxysmal atrial fibrillation (CPAF) (n = 43), cancer associated (CA) (n = 79), arteriogenic emboli (AE) (n = 522), paradoxical embolism (PE) (n = 190), and undetermined embolism (unidentified or ≥2 potential embolic sources) (UE) (n = 1120).

**Main Outcomes and Measures:**

The association between potential causes and functional outcome was evaluated in reference to cardioembolic stroke (CE) caused by major-risk cardioembolic sources after adjusting for age, sex, National Institutes of Health Stroke Scale score on admission, and reperfusion therapy using logistic regression analysis. Functional dependency (modified Rankin Scale score, 3-5) was evaluated at 3 months after onset.

**Results:**

The study enrolled 2261 patients with CE (mean [SD] age, 78.4 [10.7] years, 51.8% male) and 2163 patients with ESUS (mean [SD] age, 72.4 [12.6] years, 57.1% male). Compared with CE (median National Institutes of Health Stroke Scale score, 8 [interquartile range {IQR}, 3-17]), baseline neurological deficits did not differ in MCS (median, 7 [IQR, 2-18]), CPAF (median, 6 [IQR, 2-18]), and CA (median, 5 [IQR, 2-13]) but were less severe in AE (median, 2 [IQR, 1-4]), PE (median, 2 [IQR, 1-4]), and UE (median, 3 [IQR, 1-7]). Multivariable-adjusted odds ratios of functional dependency significantly increased in CA (3.61; 95% CI, 1.52-8.54 vs CE) but decreased in PE (0.33; 95% CI, 0.16-0.71 vs CE).

**Conclusions and Relevance:**

Potential causes are associated with poststroke outcome in patients with cryptogenic stroke. Embolic sources potentially underlying cryptogenic stroke should be considered significant variables associated with outcome.

## Key Points

**Question:**

Is functional outcome similar among potential embolic sources after cryptogenic stroke, and is it better or worse compared with that in cardioembolic stroke?

**Findings:**

Among 2261 patients with cardioembolic stroke and 2163 patients with embolic stroke of undetermined source in this multicenter hospital-based stroke registry cohort study, multivariable-adjusted odds ratios of functional dependency increased in patients with cancer-associated stroke vs cardioembolic stroke but decreased in patients with paradoxical embolism vs cardioembolic stroke.

**Meaning:**

Potential embolic sources should be considered an important variable associated with functional outcome after cryptogenic stroke.

## Teaser

This multicenter registry-based cohort study of patients hospitalized with ischemic stroke investigates whether functional outcome differs according to potential embolic sources after cryptogenic stroke.

## Introduction

Most cases of ischemic stroke result from common causes, including large artery atherosclerosis, small vessel occlusion, and cardioembolism (CE), but occasionally result from uncommon causes, such as arterial dissection and hematological disorders.^1^ Despite extensive investigation, the attributable cause of stroke cannot be specified in a proportion of patients. Nonlacunar strokes with no cardioembolic source and proximal atherosclerosis are termed *cryptogenic stroke*.^2^ The reported proportion of cryptogenic stroke among patients with ischemic stroke ranges from 23% to 40%.^3,4^

Cryptogenic stroke is assumed to be predominantly caused by thromboembolic mechanisms. To explore effective treatments for secondary prevention in cryptogenic stroke, a new clinical construct termed *embolic stroke of undetermined source* (ESUS) has been proposed by the Cryptogenic Stroke/ESUS International Working Group.^5^ Several clinical trials, such as RE-SPECT ESUS (Randomized, Double-blind, Evaluation in Secondary Stroke Prevention Comparing the Efficacy and Safety of the Oral Thrombin Inhibitor Dabigatran Etexilate vs Acetylsalicylic Acid in Patients With Embolic Stroke of Undetermined Source),^6^ the NAVIGATE ESUS (New Approach Rivaroxaban Inhibition of Factor Xa in a Global Trial vs ASA to Prevent Embolism in Embolic Stroke of Undetermined Source) trial,^7^ and the ATTICUS (Apixaban for Treatment of Embolic Stroke of Undetermined Source) randomized trial,^8^ have made initial attempts to determine the optimal antithrombotic therapy in patients with ESUS. However, the NAVIGATE ESUS trial was recently stopped early because comparable efficacy was observed between rivaroxaban and aspirin treatment arms.^9^

Cryptogenic stroke, currently termed *ESUS*, includes strokes caused by a broad spectrum of etiologies. Therefore, clinical outcomes may differ depending on potential causes. However, available data regarding clinical features in patients with ESUS are limited,^10,11^ and poststroke clinical outcomes associated with each embolic source are unclear. The present study sought to investigate whether functional outcome differs according to potential embolic sources in patients with cryptogenic stroke compared with patients with CE.

## Methods

### Study Design

We constructed a multicenter, hospital-based, prospective stroke registry (Fukuoka Stroke Registry [FSR]) in Fukuoka, Japan (University Hospital Medical Information Network Identifier UMIN0000008000). This registry included consecutive patients with acute stroke who were hospitalized within 7 days of onset in 7 participating stroke centers, including the following: Kyushu University Hospital (Fukuoka, Japan), National Hospital Organization Kyushu Medical Center (Fukuoka, Japan), National Hospital Organization Fukuoka–Higashi Medical Center (Koga, Japan), Fukuoka Red Cross Hospital (Fukuoka, Japan), St Mary’s Hospital (Kurume, Japan), Steel Memorial Yawata Hospital (Kitakyushu, Japan), and Japan Labor Health and Welfare Organization Kyushu Rosai Hospital (Kitakyushu, Japan). The institutional review committee of each hospital approved the study protocol. Written informed consent was obtained from all participants or a family member. Standardized instruments were used to collect demographic characteristics, comorbidities, laboratory data, and medical histories of the patients.^12,13^ Stroke was defined as a sudden onset of nonconvulsive and focal neurological deficit. Ischemic stroke was diagnosed using brain computed tomography or magnetic resonance imaging. In the present study, we investigated 9866 consecutive patients with acute ischemic stroke enrolled in the FSR from June 11, 2007, to May 31, 2016, in Fukuoka, Japan. We used the Strengthening the Reporting of Observational Studies in Epidemiology (STROBE) reporting guidelines for observational studies to guide the reporting of this study.

### Definition of ESUS

As cryptogenic stroke, ESUS was diagnosed based on criteria proposed by the Cryptogenic Stroke/ESUS International Working Group^5^ for nonlacunar ischemic stroke detected by computed tomography or magnetic resonance imaging in the absence of (1) luminal stenosis (≥50%) in arteries supplying the area of ischemia, (2) major-risk cardioembolic sources, and (3) other specific causes of stroke. Briefly, major subtypes of ischemic stroke were first classified into small vessel occlusion, large artery atherosclerosis, CE, and unclassified type (stroke of other determined or undetermined etiology) according to the Trial of Org 10172 in Acute Stroke Treatment (TOAST) criteria.^1^ Patients with small vessel occlusion (n = 3130), extracranial and intracranial atherosclerosis causing at least 50% luminal stenosis in arteries supplying the area of ischemia (n = 2011), and other specific uncommon causes of stroke identified (n = 301) were then excluded. Thereafter, we defined stroke due to major-risk cardioembolic sources as CE (n = 2261) (eMethods in the Supplement). Finally, we defined the remaining cryptogenic stroke as ESUS (n = 2163) (eFigure 1 in the Supplement).

### Diagnosis of Potential Embolic Sources

Potential causes of ESUS were investigated based on methods proposed by the Cryptogenic Stroke/ESUS International Working Group. All patients underwent brain computed tomography and/or magnetic resonance imaging, blood tests, chest radiography, 12-lead electrocardiogram, and electrocardiogram monitoring during the acute stage of stroke. The other diagnostic assessments for ESUS were 24-hour ambulatory Holter monitoring, echocardiography, and imaging of the proximal arteries. The frequency of each diagnostic assessment in this study is listed in eTable 1 in the Supplement. Potential embolic sources were diagnosed according to the findings of these assessments (eMethods in the Supplement) and categorized into the following 6 groups: minor-risk potential cardioembolic sources (MCS), covert paroxysmal atrial fibrillation (CPAF), cancer associated (CA), arteriogenic emboli (AE), paradoxical embolism (PE), and undetermined embolism (UE).^5^ Undetermined embolism was defined when any of the potential causes could not be identified or when more than 1 potential cause was found among 5 different causes.

### Baseline Characteristics and Poststroke Therapy

We evaluated baseline characteristics, such as age, sex, cardiovascular risk factors (hypertension, diabetes, dyslipidemia, smoking, and drinking), comorbidities (chronic kidney disease and coronary artery disease), and prestroke dependency (modified Rankin Scale [mRS] score before admission, ≥2) (eMethods in the Supplement). Neurological severity was scaled by the National Institutes of Health Stroke Scale (NIHSS) score. Reperfusion therapy included intravenous thrombolysis with recombinant tissue plasminogen activator and endovascular therapy with intra-arterial thrombolysis or mechanical thrombectomy. Antithrombotic therapy included treatments with antiplatelets (aspirin, clopidogrel bisulfate, ticlopidine hydrochloride, or cilostazol) or anticoagulants (warfarin potassium, dabigatran etexilate, rivaroxaban, apixaban, or edoxaban tosylate) prescribed at discharge.

### Clinical Outcomes

Primary outcomes were functional dependency and poor functional outcome at 3 months after onset. We defined functional dependency as an mRS score of 3 to 5 and poor functional outcome as an mRS score of 3 to 6, which included death in addition to functional dependency. Secondary outcomes were stroke recurrence and mortality assessed within 3 months of onset (eMethods in the Supplement). Stroke recurrence included ischemic and hemorrhagic strokes developing after admission for index stroke. Mortality was defined as death from any cause. The primary and secondary outcomes were also assessed at discharge for reference.

### Statistical Analysis

We compared the background characteristics, neurological severity, and rates of stroke recurrence and mortality among potential causes of ESUS by analysis of variance, Kruskal-Wallis test, χ^2^ test, or Fisher exact probability test, where appropriate. The differences between ESUS owing to each potential cause and CE were further evaluated using multiple comparison tests, such as the Steel test and the Benjamini-Hochberg procedure.

In the analysis of functional outcomes, we excluded 1170 patients whose activities of daily living were impaired (mRS score, ≥2) before stroke onset. Odds ratios (ORs) and 95% CIs of unfavorable functional outcomes, including functional dependency (mRS score, 3-5) and poor functional outcome (mRS score, 3-6), were estimated for each potential cause of ESUS in reference to CE. The multivariable logistic regression model included age, sex, NIHSS score on admission, and reperfusion therapy in consideration of the clinical relevance. The NIHSS scores on admission were categorized into 3 clinically meaningful groups (mild if 0-4, moderate if 5-14, and severe if ≥15). Six types of potential causes were simultaneously entered into the model. Sensitivity analyses were performed by entering NIHSS score as a continuous variable or additionally including prestroke mRS score of 0 or 1 in the multivariable model. Patients for whom data regarding the mRS score at 3 months were missing were excluded from the analyses of functional outcomes at 3 months. Statistical analyses were performed using JMP Pro 13 (SAS Institute Inc) and SAS, version 9.4 (SAS Institute Inc). Two-sided probability values of *P* < .05 were considered statistically significant.

## Results

### ESUS and Potential Embolic Sources

A total of 9866 patients with acute ischemic stroke were registered in the FSR between June 11, 2007, and May 31, 2016. Of these patients, 2261 patients (22.9%) (mean [SD] age, 78.4 [10.7] years; 51.8% male) were diagnosed as having CE (ie, stroke caused by major-risk sources). Diagnosis of ESUS was made in 2163 patients (21.9%) (mean [SD] age, 72.4 [12.6] years; 57.1% male). The details of potential causes that were identified in patients with ESUS are listed in Table 1. Among potential embolic sources, AE (24.1% [522 of 2163]) was most frequent, followed by MCS (9.7% [209 of 2163]), PE (8.8% [190 of 2163]), CA (3.7% [79 of 2163]), and CPAF (2.0% [43 of 2163]). Among 51.8% (1120 of 2163) of patients with UE, 9.2% (198 of 2163) had more than 1 potential cause of different etiologies, and potential causes could not be identified in 42.6% (922 of 2163). There were significant differences in the background characteristics between CE and ESUS (Table 2).

**Table 1.** Potential Embolic Sources Based on Criteria Proposed by the Cryptogenic Stroke/ESUS International Working Group

Potential Embolic Source	No. (%) of Cases (n = 2163)
Minor-risk potential cardioembolic sources^a^	209 (9.7)
Mitral valve	
Myxomatous valvulopathy with prolapse	2 (0.1)
Mitral annular calcification	7 (0.3)
Aortic valve	
Aortic valve stenosis	13 (0.6)
Calcific aortic valve	4 (0.2)
Nonatrial fibrillation atrial dysrhythmias and stasis	
Atrial asystole and sick sinus syndrome	43 (2.0)
Atrial high-rate episodes	32 (1.5)
Atrial appendage stasis with reduced flow velocities or spontaneous echodensities	21 (1.0)
Atrial structural abnormalities	
Atrial septal aneurysm	13 (0.6)
Chiari network	1 (0.0)
Left ventricle	
Moderate systolic or diastolic dysfunction, global or regional	80 (3.7)
Ventricular noncompaction	0
Endomyocardial fibrosis	0
Covert paroxysmal atrial fibrillation	43 (2.0)
Cancer associated	79 (3.7)
Covert nonbacterial thrombotic endocarditis	79 (3.7)
Tumor emboli from occult cancer	0
Arteriogenic emboli^a^	522 (24.1)
Aortic arch atherosclerotic plaques	520 (24.0)
Carotid artery nonstenotic plaques with ulceration	4 (0.2)
Paradoxical embolism	190 (8.8)
Patent foramen ovale	168 (7.8)
Atrial septal defect	10 (0.5)
Pulmonary arteriovenous fistula	12 (0.6)
Undetermined embolism	1120 (51.8)
Multiple embolic sources^b^	198 (9.2)
Undetermined source^c^	922 (42.6)

Abbreviation: ESUS, embolic stroke of undetermined source.

^a^ Patients with 2 or more potential embolic sources of minor-risk potential cardioembolic sources or arteriogenic emboli were included in the minor-risk potential cardioembolic sources or arteriogenic emboli group, respectively.

^b^ Patients with 2 or more potential embolic sources of different etiologies were categorized as patients with multiple embolic sources in the undetermined embolism group.

^c^ Patients whose potential cause could not be identified were categorized as patients with undetermined source in the undetermined embolism group.

**Table 2.** Clinical Characteristics of Patients With CE and ESUS

Characteristic	CE (n = 2261)	ESUS (n = 2163)	*P* Value	ESUS						*P* Value
				MCS (n = 209)	CPAF (n = 43)	CA (n = 79)	AE (n = 522)	PE (n = 190)	UE (n = 1120)	
Age, mean (SD), y	78.4 (10.7)	72.4 (12.6)	<.001	76.9 (12.4)	75.2 (11.2)	76.6 (10.7)	74.4 (9.7)^a^	64.8 (13.6)^a^	71.5 (13.2)^a^	<.001
Men, No. (%)	1171 (51.8)	1235 (57.1)	<.001	101 (48.3)	19 (44.2)	40 (50.6)	358 (68.6)^a^	112 (58.9)	605 (54.0)	<.001
Cardiovascular risk factors, No. (%)										
Hypertension	1735 (76.7)	1647 (76.1)	.64	167 (79.9)	31 (72.1)	52 (65.8)^a^	445 (85.2)^a^	108 (56.8)^a^	844 (75.4)	<.001
Diabetes	501 (22.2)	636 (29.4)	<.001	60 (28.7)	11 (25.6)	15 (19.0)	181 (34.7)^a^	49 (25.8)	320 (28.6)^a^	.02
Dyslipidemia	815 (36.0)	1112 (51.4)	<.001	110 (52.6)^a^	18 (41.9)	23 (29.1)	309 (59.2)^a^	89 (46.8)^a^	563 (50.3)^a^	<.001
Smoking	985 (43.6)	1128 (52.1)	<.001	82 (39.2)	19 (44.2)	32 (40.5)	345 (66.1)^a^	84 (44.2)	566 (50.5)^a^	<.001
Drinking	700 (31.0)	673 (31.1)	.91	51 (24.4)	12 (27.9)	18 (22.8)	185 (35.4)	67 (35.3)	340 (30.4)	.02
Comorbidity, No. (%)										
Chronic kidney disease	1278 (56.5)	957 (44.2)	<.001	109 (52.2)	25 (58.1)	40 (50.6)	245 (46.9)^a^	52 (27.4)^a^	486 (43.4)^a^	<.001
Coronary artery disease	399 (17.6)	343 (15.9)	.11	79 (37.8)^a^	7 (16.3)	6 (7.6)^a^	101 (19.3)	13 (6.8)^a^	137 (12.2)^a^	<.001
Prestroke dependency, No. (%)	679 (30.0)	491 (22.7)	<.001	74 (35.4)	10 (23.3)	25 (31.6)	104 (19.9)^a^	17 (8.9)^a^	261 (23.3)^a^	<.001
NIHSS score on admission										
Mean (SD)	10.4 (8.7)	5.3 (6.8)	<.001	10.0 (9.3)	10.0 (9.3)	8.1 (7.2)	3.3 (3.9)^a^	3.4 (5.3)^a^	5.4 (6.8)^a^	<.001
Median (IQR)	8 (3-17)	3 (1-6)	NA	7 (2-18)	6 (2-18)	5 (2-13)	2 (1-4)^a^	2 (1-4)^a^	3 (1-7)^a^	NA
Reperfusion therapy, No. (%)	476 (21.1)	202 (9.3)	<.001	48 (23.0)	5 (11.6)	4 (5.1)^a^	28 (5.4)^a^	21 (11.1)^a^	96 (8.6)^a^	<.001
Intravenous thrombolysis	442 (19.5)	190 (8.8)	<.001	47 (22.5)	5 (11.6)	4 (5.1)^a^	27 (5.2)^a^	17 (8.9)^a^	90 (8.0)^a^	<.001
Endovascular therapy	99 (4.4)	31 (1.4)	<.001	5 (2.4)	1 (2.3)	0	1 (0.2)^a^	6 (3.2)	18 (1.6)^a^	.008
Antithrombotic therapy, No./total No. (%)^b^										
Antiplatelets	319/2134 (14.9)	1475/2123 (69.5)	<.001	102/205 (49.8)^a^	6/43 (14.0)	9/65 (13.8)	476/521 (91.4)^a^	96/189 (50.8)^a^	786/1100 (71.5)^a^	<.001
Anticoagulants	1944/2134 (91.1)	669/2123 (31.5)	<.001	145/205 (70.7)^a^	37/43 (86.0)	28/65 (43.1)^a^	57/521 (10.9)^a^	95/189 (50.3)^a^	307/1100 (27.9)^a^	<.001

Abbreviations: AE, arteriogenic emboli; CA, cancer associated; CE, cardioembolic stroke; CPAF, covert paroxysmal atrial fibrillation; ESUS, embolic stroke of undetermined source; IQR, interquartile range; MCS, minor-risk potential cardioembolic sources; NA, not applicable; NIHSS, National Institutes of Health Stroke Scale; PE, paradoxical embolism; UE, undetermined embolism.

^a^ *P* < .05 vs CE by multiple comparisons.

^b^ Number and percentage of patients receiving antithrombotic therapy at discharge after excluding patients who died during hospitalization.

### Baseline Characteristics and Poststroke Therapy

Significant differences in the background characteristics were found according to the potential causes of ESUS (Table 2). Patients with AE, PE, or UE were younger than those with CE, and the proportion of potential causes varied depending on age (eFigure 2A in the Supplement). Male sex was more common in the AE group than in the CE group. Frequencies of cardiovascular risk factors significantly differed according to potential causes. Compared with the CE group, hypertension was higher in the AE group but lower in the CA and PE groups; diabetes was higher in the AE and UE groups; dyslipidemia was higher in the MCS, AE, PE, and UE groups; smoking was higher in the AE and UE groups; chronic kidney disease was lower in the AE, PE, and UE groups; and coronary artery disease was higher in the MCS group but lower in the CA, PE, and UE groups. Prestroke dependency was less prevalent among the AE, PE, and UE groups compared with the CE group.

Reperfusion therapy was similarly performed between the MCS or CPAF group and the CE group. However, patients with CA, AE, PE, or UE underwent reperfusion therapy less frequently than those with CE. Antiplatelet therapy was more frequently prescribed in the MCS, AE, PE, and UE groups than in the CE group. Patients with any potential causes except CPAF underwent anticoagulation therapy less frequently than patients with CE.

### Stroke Severity

**Figure 1.**
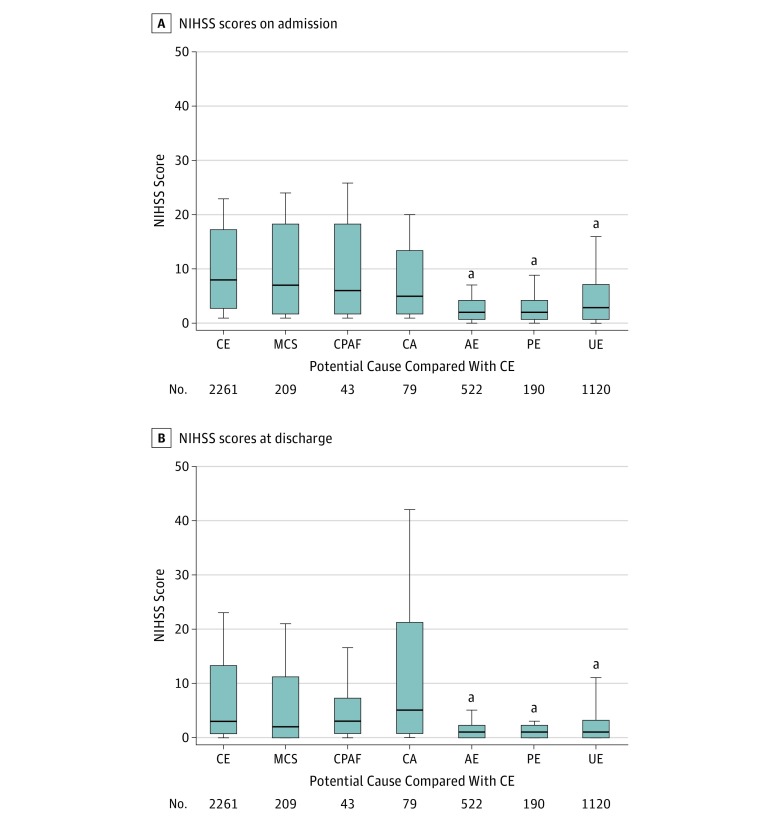
Neurological Severity The National Institutes of Health Stroke Scale (NIHSS) scores on admission (A) and at discharge (B) are shown according to each potential cause compared with cardioembolic stroke (CE). The NIHSS score can range from 1 to 42 and measures neurological severity categorized into 3 clinically meaningful groups (mild if 0-4, moderate if 5-14, and severe if ≥15). In patients who died during hospitalization, the NIHSS score at discharge was assigned the maximum score of 42. The box indicates ranges between lower quartile score and upper quartile score, and the horizontal line in the box represents the median score. Lower and upper vertical bars indicate the 10th and 90th percentiles, respectively. AE indicates arteriogenic emboli; CA, cancer associated; CPAF, covert paroxysmal atrial fibrillation; MCS, minor-risk potential cardioembolic sources; PE, paradoxical embolism; and UE, undetermined embolism. ^a^Statistically significant at *P* < .05 vs CE by multiple comparisons.

Neurological severity significantly differed on admission and at discharge among potential causes in patients with ESUS. On admission, neurological symptoms were less severe in the AE (median NIHSS score, 2; interquartile range [IQR], 1-4), PE (median score, 2; IQR, 1-4), and UE (median score, 3; IQR, 1-7) groups than in the CE group (median score, 8; IQR, 3-17), whereas symptoms were not significantly different between the MCS (median score, 7; IQR, 2-18), CPAF (median score, 6; IQR, 2-18), and CA (median score, 5; IQR, 2-13) groups and the CE group (Table 2 and Figure 1A). As a result, the proportion of AE and PE decreased as stroke became severe (eFigure 2B in the Supplement). At discharge, neurological symptoms tended to be less severe in the AE (median NIHSS score, 1; IQR, 0-2), PE (median score, 1; IQR, 0-2), and UE (median score, 1; IQR, 0-3) groups compared with the CE group (median score, 3; IQR, 1-13) (Figure 1B).

### Primary Outcomes

**Figure 2.**
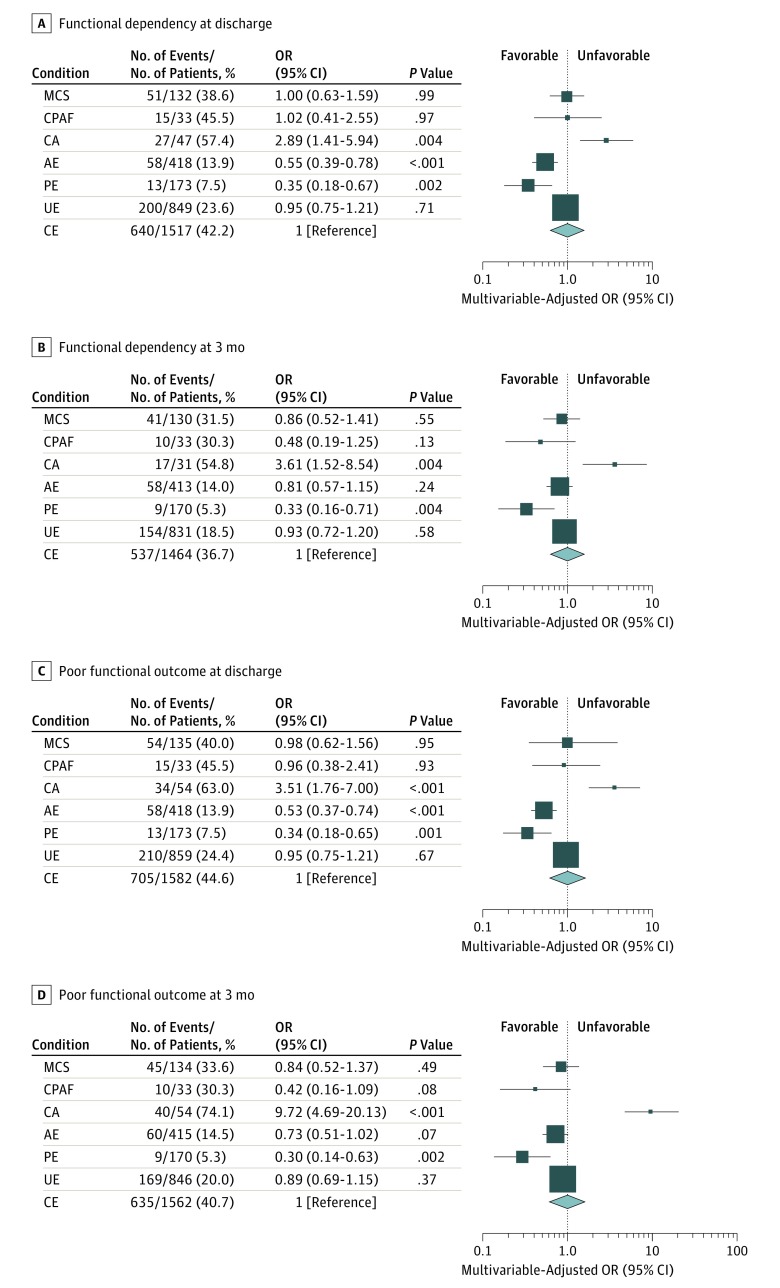
Functional Outcomes Functional dependency (A and B) and poor functional outcome (C and D) in embolic stroke of undetermined source are shown according to each potential cause compared with cardioembolic stroke (CE). Functional outcomes were evaluated at discharge (A and C) and at 3 months of stroke onset (B and D). Odds ratio (OR) (square) and 95% CI (bars) of functional outcomes are shown for each potential cause with reference to CE (diamond). The multivariable model included age, sex, National Institutes of Health Stroke Scale score (measuring neurological severity) on admission (mild if 0-4, moderate if 5-14, and severe if ≥15), and reperfusion therapy. The sizes of squares or diamonds are proportional to the sizes of the subgroups of each potential cause. Patients who died during hospitalization or within 3 months were excluded from the analysis for functional dependency. Patients who were lost to follow-up at 3 months were also excluded from the analysis for functional outcome at 3 months. AE indicates arteriogenic emboli; CA, cancer associated; CPAF, covert paroxysmal atrial fibrillation; MCS, minor-risk potential cardioembolic sources; PE, paradoxical embolism; and UE, undetermined embolism.

We compared poststroke functional outcomes according to the potential causes of ESUS after excluding 1170 patients with prestroke dependency. At discharge, the multivariable-adjusted OR of functional dependency (mRS score, 3-5) significantly decreased in the AE (OR, 0.55; 95% CI, 0.39-0.78) and PE (OR, 0.35; 95% CI, 0.18-0.67) groups but increased in the CA group (OR, 2.89; 95% CI, 1.41-5.94) compared with the CE group (Figure 2A). At 3 months after onset, the OR of functional dependency was still low in the PE group (OR, 0.33; 95% CI, 0.16-0.71) but was high in the CA group (OR, 3.61; 95% CI, 1.52-8.54) (Figure 2B).

Similar trends were maintained in the association between the potential causes of ESUS and poor functional outcomes (mRS score, 3-6) at discharge (Figure 2C) and at 3 months after onset (Figure 2D). These associations were still found even when we entered the NIHSS score into the multivariable model as a continuous variable or when we additionally adjusted for prestroke mRS score (eTable 2 and eTable 3 in the Supplement).

### Secondary Outcomes

**Figure 3.**
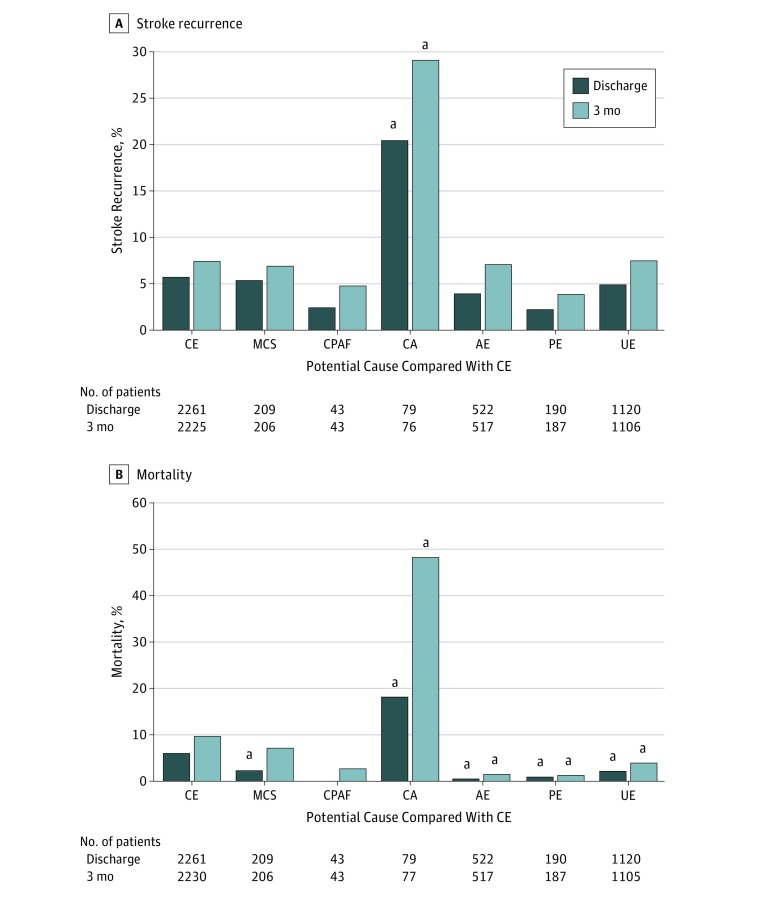
Stroke Recurrence and Mortality Rates of stroke recurrence (A) and mortality (B) are shown according to each potential cause compared with cardioembolic stroke (CE). Patients whose data regarding stroke recurrence or mortality at 3 months were missing were excluded from the analysis for the respective adverse events at 3 months. The number of patients in each group is shown below the graph. AE indicates arteriogenic emboli; CA, cancer associated; CPAF, covert paroxysmal atrial fibrillation; MCS, minor-risk potential cardioembolic sources; PE, paradoxical embolism; and UE, undetermined embolism. ^a^Statistically significant at *P* < .05 vs CE by multiple comparisons.

Although the rate of stroke recurrence did not differ between patients with CE and overall ESUS, stroke recurrence significantly differed among the potential causes of ESUS (eTable 4 in the Supplement). Stroke recurrence in the CA group (20.3% [16 of 79] at discharge and 28.9% [22 of 76] at 3 months) was more frequent compared with that in the CE group (5.6% [126 of 2261] at discharge and 7.3% [162 of 2225] at 3 months) (Figure 3A).

Mortality was significantly lower in overall ESUS (1.8% [40 of 2163] at discharge and 4.6% [98 of 2135] at 3 months) than in patients with CE (5.6% [127 of 2261] at discharge and 9.4% [209 of 2230] at 3 months (eTable 4 in the Supplement). There was also a significant difference in mortality among potential causes of ESUS. In-hospital mortality was significantly higher in the CA group (17.7% [14 of 79]) but was lower in the MCS (1.9% [4 of 209]), AE (0.2% [1 of 522]), PE (0.5% [1 of 190]), and UE (1.8% [20 of 1120]) groups than in the CE group (5.6% [127 of 2261]) (Figure 3B). Mortality at 3 months was still higher in the CA group (48.1% [37 of 77]) but lower in the AE (1.0% [5 of 517]), PE (1.1% [2 of 187]), and UE (3.5% [39 of 1105]) groups.

## Discussion

The present study revealed several major findings. Functional outcomes at 3 months after onset were worse in the CA group but better in the PE group than in the CE group, even after adjusting for potential confounding factors. Compared with the CE group, early stroke recurrence within 3 months was more frequent in the CA group; 3-month mortality was higher in the CA group but lower in the AE, PE, and UE groups. These findings indicate that the poststroke clinical outcomes in patients with cryptogenic stroke (ESUS) are not the same as those in CE according to potential embolic sources. Poststroke therapy may need to be personalized considering the potential causes in patients with cryptogenic stroke.

### Prevalence of Potential Embolic Sources

The frequency of ESUS reported in previous studies^10,14,15,16,17^ has ranged from 9% to 25%. The present study revealed that 21.9% (2163 of 9866) of acute ischemic stroke cases were diagnosed as ESUS, indicating that ESUS is not uncommon among ischemic strokes. The reported prevalence of potential causes has also varied between studies, with values of 18% for MCS,^18^ 10% to 19% for CPAF,^19,20^ 20% to 38% for CA,^21,22^ 28% to 47% for AE,^23,24^ and 40% for patent foramen ovale.^25^ In a case series of 321 patients with ESUS in Korea,^15^ PE was most prevalent, followed by CPAF and AE. In the Athens Stroke Registry^10^ among 275 patients with ESUS, MCS and CPAF were predominant causes (>80%). In contrast, we found that a considerable proportion of ESUS was caused by MCS, AE, and PE and that CPAF and CA were rare but not negligible potential causes of ESUS. This diversity is likely to be due to differences in diagnostic assessment among the reports.

### Primary Outcomes

A median NIHSS score at onset of 5 in patients with ESUS was reported in the Athens Stroke Registry,^14^ while a median score of 4 was reported in the ESUS Global Registry.^16^ Four studies among 1772 patients with ESUS had a mean NIHSS score of 5 in a systematic review.^17^ The present study revealed a median NIHSS score of 3 in all patients with ESUS, indicating that patients with ESUS mostly exhibited minor stroke. However, detailed analysis further revealed that stroke severity in the MCS, CPAF, and CA groups was comparable to that in the CE group, whereas strokes due to AE, PE, or UE were less severe than those due to CE.

The present study demonstrated that 3-month functional outcomes were better in the PE group but worse in the CA group compared with the CE group, even after controlling for baseline neurological severity or reperfusion therapy. There are several plausible explanations for this diversity. First, the size and nature of thrombi generated by each potential cause may vary, leading to different clinical courses. Second, the background characteristics and poststroke therapy may have influenced poststroke recovery. Specifically, younger age may have led to better outcomes, particularly in patients with PE. Third, early stroke recurrence and the poststroke adverse events may have influenced poststroke outcome. Although stroke is caused equally by thromboembolic mechanisms, ESUS can result in diverse clinical outcomes according to potential embolic sources.

### Secondary Outcomes

In patients with ESUS, the rate of stroke recurrence per year was reported to be 5.4% in the Instituto Nacional de Neurología y Neurocirugía Stroke Registry^26^ and 6.8% in the Athens Stroke Registry.^14^ The annualized recurrent stroke rate was estimated as 4.5% per year from 5 studies of 1605 patients with ESUS in a systematic review.^17^ In the present study, early stroke recurrence in all patients with ESUS was more frequent than expected (in-hospital recurrence of 4.9% [106 of 2163] and 3-month recurrence of 7.6% [162 of 2135]). Furthermore, the cause-specific recurrence rates significantly differed: patients with CA stroke had a high risk of early stroke recurrence among patients with ESUS.

Mortality per 100 patient-years among patients with ESUS was reported as 8.2 in the Athens Stroke Registry.^14^ Pooled data sets of 11 stroke registries from Europe and the United States revealed mortality rates of 3.48 in women and 3.98 in men per 100 patient-years.^11^ In the present study, in-hospital and 3-month mortality rates in all patients with ESUS were 1.8% (40 of 2163) and 4.6% (98 of 2135), respectively. However, cause-specific mortality rates significantly differed: the mortality rate in patients with CA stroke was high, as expected, whereas mortality rates among patients with AE, PE, and UE were low compared with mortality rates in patients with CE. However, the present study included both patients with known cancers and patients with occult cancers diagnosed as part of the stroke evaluation. Therefore, mortality may have been biased by the presence of cancer in the present study.

### Limitations

The present study had several limitations that should be considered. Stroke subtypes were retrospectively reassigned according to the criteria proposed by the Cryptogenic Stroke/ESUS International Working Group, potentially leading to diagnostic bias. Patients with ESUS did not always receive all of the diagnostic testing necessary to meet the definition of these categories; therefore, other potential causes may have existed in patients with UE. Participating hospitals were 7 stroke centers in the FSR to which patients with stroke were sometimes referred because of unidentified causes, potentially leading to a high proportion of cryptogenic stroke. We excluded from the analyses patients whose data were missing at 3 months (eTable 5 in the Supplement), although the influence may have been minimal. The number of study participants was low for estimating accurate event rates. Because only Japanese patients were recruited in the present study, collaboration and validation are required in other populations and among different racial/ethnic groups.

## Conclusions

Potential embolic sources were associated with functional outcome in patients with ESUS. The potential cause should be considered an important variable associated with functional outcome after cryptogenic stroke.

## Notes

Supplement. **eMethods.** Supplemental Methods **eFigure 1.** Flow Chart of Patient Selection Process **eFigure 2.** Proportion of Potential Causes According to Age and Stroke Severity **eTable 1.** Diagnostic Assessment of Potential Causes **eTable 2.** Association of Embolic Sources With Functional Dependency in the Sensitivity Analysis **eTable 3.** Association of Embolic Sources With Poor Functional Outcome in the Sensitivity Analysis **eTable 4.** Adverse Events During Hospitalization and at 3 Months **eTable 5.** Background Characteristics of Patients With Complete and Incomplete Data on 3-Month Outcome
