# Supplementary material for: Association of Embolic Sources With Cause-Specific Functional Outcomes Among Adults With Cryptogenic Stroke
Source: JAMA Netw Open. 2018 Sep 28;1(5):e182953. doi: 10.1001/jamanetworkopen.2018.2953 (PMC6324510; doi:10.1001/jamanetworkopen.2018.2953)
Supplement: Supplement. — eMethods. Supplemental Methods eFigure 1. Flow Chart of Patient Selection Process eFigure 2. Proportion of Potential Causes According to Age and Stroke Severity eTable 1. Diagnostic Assessment of Potential Causes eTable 2. Association of Embolic Sources With Functional Dependency in the Sensitivity Analysis eTable 3. Association of Embolic Sources With Poor Functional Outcome in the Sensitivity Analysis eTable 4. Adverse Events During Hospitalization and at 3 Months eTable 5. Background Characteristics of Patients With Complete and Incomplete Data on 3-Month Outcome [file jamanetwopen-1-e182953-s001.pdf]

Supplementary Online Content

Kiyuna F, Sato N, Matsuo R, et al; Fukuoka Stroke Registry Investigators. Association of embolic sources with cause-specific functional outcomes among adults with cryptogenic stroke. *JAMA Netw Open*. 2018;1(5):e182953.
doi:10.1001/jamanetworkopen.2018.2953

eMethods. Supplemental Methods

eFigure 1. Flow Chart of Patient Selection Process

eFigure 2. Proportion of Potential Causes According to Age and Stroke Severity

eTable 1. Diagnostic Assessment of Potential Causes

eTable 2. Association of Embolic Sources With Functional Dependency in the Sensitivity Analysis

eTable 3. Association of Embolic Sources With Poor Functional Outcome in the Sensitivity Analysis

eTable 4. Adverse Events During Hospitalization and at 3 Months

eTable 5. Background Characteristics of Patients With Complete and Incomplete Data on 3-Month Outcome

This supplementary material has been provided by the authors to give readers additional information about their work.

© 2018 Kiyuna F et al. *JAMA Network Open*.

eMethods. Supplemental Methods

Stroke subtype

Stroke subtypes were categorized into small-vessel occlusion, large-artery atherosclerosis, cardioembolism, and other subtypes based on admission based on the classification of the Trial of Org 10172 in Acute Stroke Treatment (TOAST) study.[1] However, TOAST classification considers not only stroke caused by high-risk sources but also stroke due to medium-risk sources as cardioembolic stroke.[1] In contrast, the Cryptogenic Stroke/ESUS International Working Group defines cardioembolic stroke only when patients have major-risk sources of cardioembolism.[2] Accordingly, the current study categorized only cardioembolic stroke due to major-risk cardiac sources as cardioembolic stroke. Large-artery atherosclerosis was defined as extracranial or intracranial atherosclerosis causing more than 50% luminal stenosis in arteries supplying the area of ischemia. Small-vessel occlusion was defined as a subcortical brain infarct smaller than or equal to 1.5 cm in the distribution of small, penetrating cerebral arteries.

Potential embolic sources

Potential embolic causes were categorized into six groups, minor-risk potential cardioembolic sources, covert paroxysmal atrial fibrillation, cancer-associated, arteriogenic emboli, paradoxical embolism, and undetermined embolism, based on the criteria proposed by Cryptogenic Stroke/ESUS International Working Group.[2] Minor-risk potential cardioembolic sources included medium-risk sources in the TOAST classification[1] and other potential cardiac sources proposed by the Working Group.[2] Cardiac sources were diagnosed by echocardiography according to guidelines from the American Society of Echocardiography[3] and/or other diagnostic tools, as needed. Covert paroxysmal atrial fibrillation was defined when doctors suspected underlying paroxysmal atrial fibrillation by clinical symptoms, such as palpitation and irregular heartbeat, or other findings, such as frequent supraventricular extrasystoles, in the absence of direct evidence of atrial fibrillation by electrocardiogram. Cancer-associated stroke was defined based on the findings of non-bacterial thrombotic endocarditis in the absence of other potential causes. Non-bacterial thrombotic endocarditis was diagnosed when vegetations were detected by echocardiography in patients with advanced malignancies in the absence of systemic infection. Definite non-bacterial thrombotic endocarditis was classified as cardioembolism; however, covert non-bacterial thrombotic endocarditis was regarded as a potential cause associated with cancer. Covert non-bacterial thrombotic endocarditis was diagnosed by characteristic clinical manifestations compatible with its diagnosis, including negative blood cultures, absence of clinical signs of infection, presence of hypercoagulable state, and supportive brain imaging evidence such as small, multiple and scattered lesions in the widely distributed territories. Arteriogenic emboli included aortic arch atherosclerotic plaques and carotid artery non-stenotic plaques with ulcerated or irregular surface. Aortic arch atherosclerotic plaques were defined as aortic arch atheroma with complex aortic plaques evaluated by transesophageal echocardiography or computed tomography angiography. Complex aortic plaques included plaques ≥4 mm thickness, ulceration, or mobile plaques. Paradoxical embolism included patent foramen ovale, atrial septal defect, and pulmonary arteriovenous fistula. Patent foramen ovale was diagnosed when microbubbles were observed passing through the foramen ovale from the right to left atrium during the Valsalva maneuver after intravenous injection of the contrast agent. Pulmonary arteriovenous fistula was diagnosed based on a right-to-left shunting and radiological findings of chest computed tomography or pulmonary angiogram suggesting the presence of pulmonary arteriovenous fistula. Undetermined embolism was defined when no attributable cause for stroke could be specified despite these investigations, or when more than one potential cause was identified among these five potential causes.

Baseline characteristics

Definitions of clinical characteristics were the same as those previously described.[4,5] Cardiovascular risk factors were defined as follows: hypertension: systolic blood pressure ≥140 mmHg or diastolic blood pressure ≥90 mmHg in the chronic stage, or a previous history of hypertension; diabetes mellitus: the diagnostic criteria of the Japan Diabetes Society[6] in the chronic stage or a medical history of diabetes; dyslipidemia: low-density lipoprotein cholesterol ≥3.62 mmol/L, high-density lipoprotein cholesterol <1.03 mmol/L, or triglycerides ≥1.69 mmol/L, or a history of antihypercholesterolemic medication; smoking: previous or current cigarette smoking; drinking: habitual consumption of alcoholic beverages. Chronic kidney disease was defined as an estimated glomerular filtration rate of less than 60 ml/min/1.73m^2.[7] Coronary artery diseases included a history of angina

© 2018 Kiyuna F et al. *JAMA Network Open*.

pectoris, coronary revascularization, myocardial infarction, or bypass surgery for coronary artery diseases. Previous stroke included both ischemic and hemorrhagic stroke. We obtained the data on pre-stroke functional status on admission from participants or their family members, and pre-stroke dependency was defined as a modified Rankin scale score ≥2 before admission.

Clinical outcomes

The National Institutes of Health Stroke Scale and modified Rankin scale were assessed by trained stroke neurologists during hospitalization. The modified Rankin scale at the 3-month time point was evaluated by stroke neurologists or trained and certified research nurses who were blinded to the baseline data in person or through telephone assessment. The outcomes were evaluated on the basis of a standardized structured questionnaire that had been validated in a previous study to minimize inter-rater variability.[8] All events were reviewed by the event adjudication committee members.[9]

eReferences

1. Adams HP, Jr., Bendixen BH, Kappelle LJ, et al. Classification of subtype of acute ischemic stroke. Definitions for use in a multicenter clinical trial. *Stroke.* 1993;24(1):35-41.
2. Hart RG, Diener H-C, Coutts SB, et al. Embolic strokes of undetermined source: the case for a new clinical construct. *Lancet Neurol.* 2014;13(4):429-438.
3. Saric M, Armour AC, Arnaout MS, et al. Guidelines for the use of echocardiography in the evaluation of a cardiac source of embolism. *J Am Soc Echocardiogr.* 2016;29(1):1-42.
4. Kamouchi M, Matsuki T, Hata J, et al. Prestroke glycemic control is associated with the functional outcome in acute ischemic stroke: the Fukuoka Stroke Registry. *Stroke.* 2011;42(10):2788-2794.
5. Kumai Y, Kamouchi M, Hata J, et al. Proteinuria and clinical outcomes after ischemic stroke. *Neurology.* 2012;78(24):1909-1915.
6. Seino Y, Nanjo K, Tajima N, et al. Report of the committee on the classification and diagnostic criteria of diabetes mellitus. *J Diabetes Investig.* 2010;1(5):212-228.
7. Matsuo S, Imai E, Horio M, et al. Revised equations for estimated GFR from serum creatinine in Japan. *Am J Kidney Dis.* 2009;53(6):982-992.
8. Shinohara Y, Minematsu K, Amano T, Ohashi Y. Modified Rankin scale with expanded guidance scheme and interview questionnaire: interrater agreement and reproducibility of assessment. *Cerebrovasc Dis.* 2006;21(4):271-278.
9. Ogata T, Matsuo R, Kiyuna F, et al. Left atrial size and long-term risk of recurrent stroke after Acute ischemic stroke in patients with nonvalvular atrial fibrillation. *J Am Heart Assoc.* 2017;6(8).

© 2018 Kiyuna F et al. *JAMA Network Open*.

eFigure 1. Flow Chart of Patient Selection Process

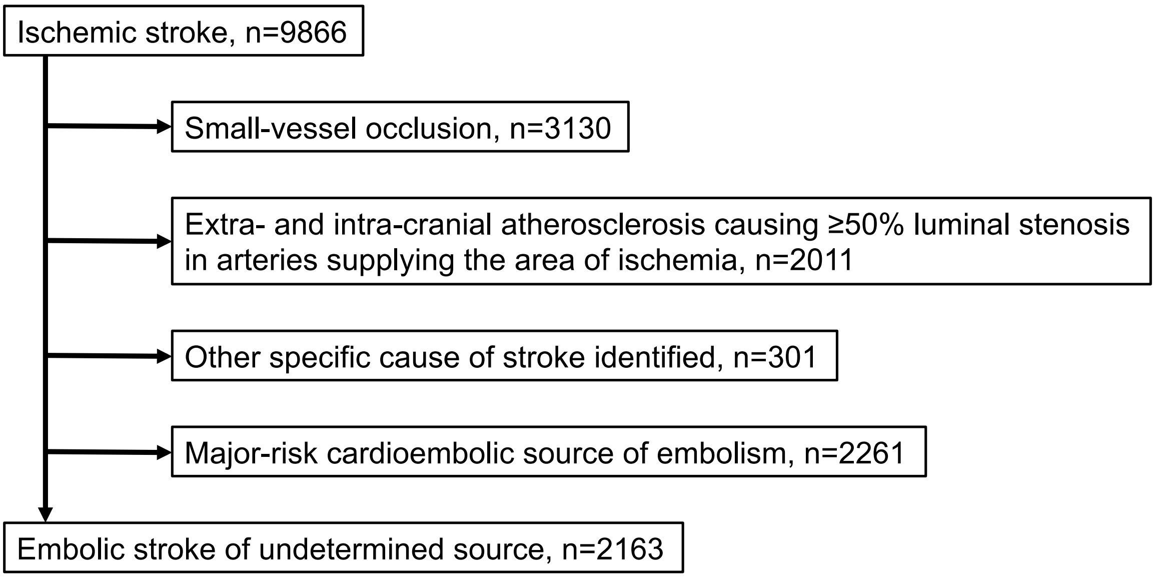

Stroke of other determined etiology included arterial dissection (n = 181), moyamoya disease (n = 19), cerebral venous thrombosis (n = 17), antiphospholipid syndrome (n = 12), hematologic disorder (n = 23), angiitis (n = 7), and other (n = 42).

© 2018 Kiyuna F et al. *JAMA Network Open.*

eFigure 2. Proportion of Potential Causes According to Age and Stroke Severity

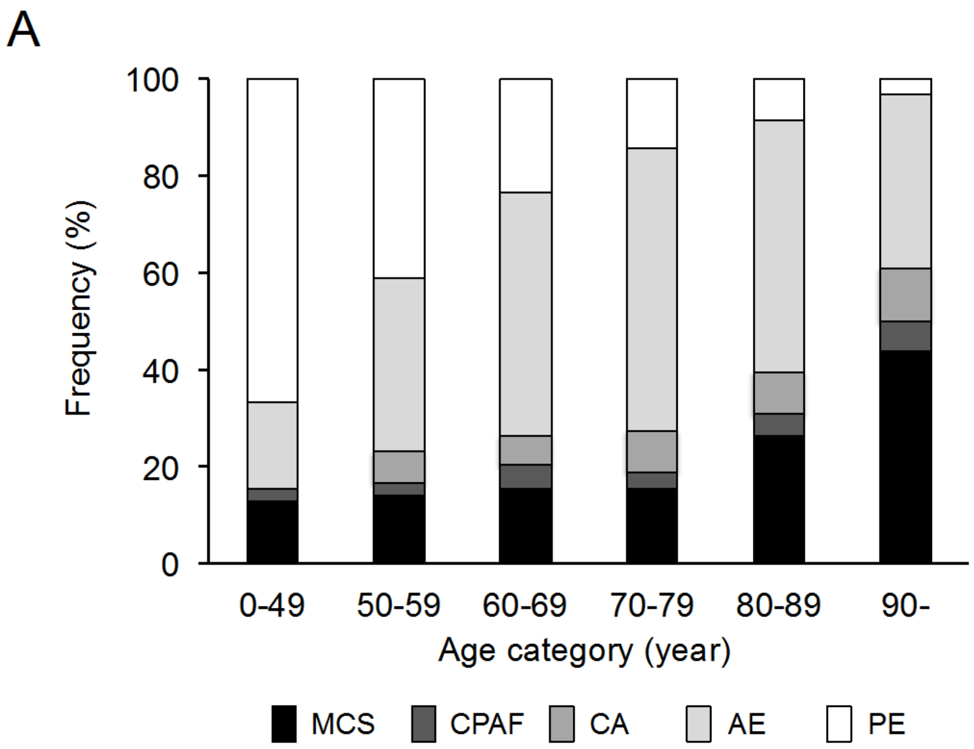

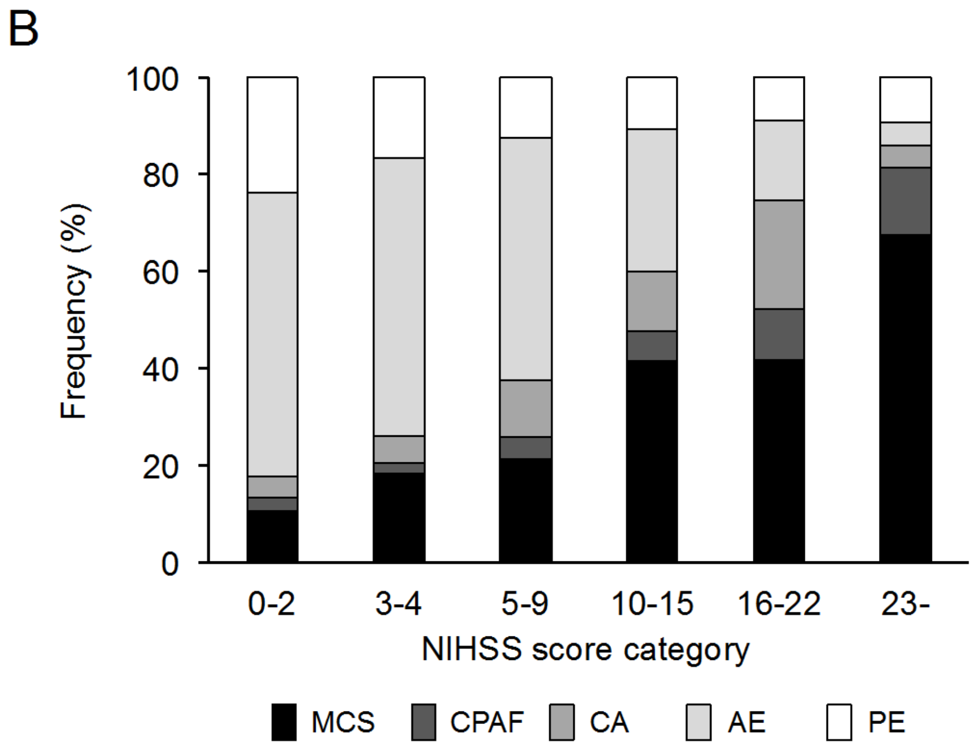

MCS: minor-risk potential cardioembolic sources, CPAF: covert paroxysmal atrial fibrillation, CA: cancer-associated, AE: arteriogenic emboli, PE: paradoxical embolism. Proportion of each potential embolic source is shown according to age category (A) or baseline stroke severity (B). Stroke severity was scaled by National Institutes of Health Stroke Scale (NIHSS) score and categorized into six groups.

© 2018 Kiyuna F et al. *JAMA Network Open*.

eTable 1. Diagnostic Assessment of Potential Causes

Brain imaging	
Brain CT	1524 (70.5)
Brain MR imaging	2123 (98.2)
ECG	
12-lead ECG	2163 (100.0)
Continuous ECG monitoring	2163 (100.0)
24-hour ambulatory Holter monitoring	2017 (93.3)
Echocardiography	
Transthoracic echocardiography	2065 (95.5)
Transesophageal echocardiography	1272 (58.8)
Imaging of both extracranial and intracranial arteries	
Carotid ultrasonography	2065 (95.5)
Intracranial MR angiography	2115 (97.8)
Extracranial MR angiography	602 (27.8)
CT angiography	367 (17.0)
Conventional angiography	75 (3.5)

CT: computed tomography, MR: magnetic resonance, ECG: electrocardiogram. Data are expressed as numbers of patients (%).

© 2018 Kiyuna F et al. *JAMA Network Open*.

eTable 2. Association of Embolic Sources With Functional Dependency in the Sensitivity Analysis

		Age- and sex-adjusted				Model 1				Model 2		
	Event (%)	OR	(95% CI)	P		OR	(95% CI)	P		OR	(95% CI)	P
Discharge												
CE, n = 1517	640 (42.2)	1.00	(reference)			1.00	(reference)			1.00	(reference)	
MCS, n = 132	51 (38.6)	0.96	(0.66–1.40)	0.83		0.99	(0.61–1.62)	0.98		1.01	(0.63–1.61)	0.97
CPAF, n = 33	15 (45.5)	1.22	(0.59–2.50)	0.59		1.02	(0.40–2.63)	0.96		1.06	(0.43–2.66)	0.90
CA, n = 47	27 (57.4)	2.09	(1.14–3.82)	0.02		3.44	(1.73–6.84)	<0.001		2.70	(1.31–5.58)	0.007
AE, n = 418	58 (13.9)	0.24	(0.18–0.32)	<0.001		0.63	(0.45–0.88)	0.008		0.55	(0.39–0.78)	<0.001
PE, n = 173	13 (7.5)	0.16	(0.09–0.29)	<0.001		0.34	(0.17–0.68)	0.002		0.34	(0.18–0.66)	0.001
UE, n = 849	200 (23.6)	0.53	(0.43–0.64)	<0.001		1.01	(0.79–1.29)	0.92		0.96	(0.75–1.22)	0.74
3 months												
CE, n = 1464	537 (36.7)	1.00	(reference)			1.00	(reference)			1.00	(reference)	
MCS, n = 130	41 (31.5)	0.89	(0.59–1.33)	0.56		0.82	(0.49–1.39)	0.47		0.88	(0.53–1.44)	0.60
CPAF, n = 33	10 (30.3)	0.77	(0.35–1.69)	0.52		0.44	(0.16–1.25)	0.12		0.50	(0.19–1.32)	0.16
CA, n = 31	17 (54.8)	2.49	(1.18–5.26)	0.02		4.43	(1.92–10.20)	<0.001		3.27	(1.35–7.91)	0.008
AE, n = 413	58 (14.0)	0.32	(0.23–0.43)	<0.001		0.89	(0.63–1.26)	0.52		0.81	(0.57–1.16)	0.25
PE, n = 170	9 (5.3)	0.15	(0.07–0.29)	<0.001		0.33	(0.15–0.72)	0.005		0.31	(0.14–0.68)	0.003
UE, n = 831	154 (18.5)	0.51	(0.41–0.63)	<0.001		0.98	(0.75–1.28)	0.88		0.94	(0.72–1.22)	0.63

OR: odds ratio, CI: confidence interval, CE: cardioembolic stroke, MCS: minor-risk potential cardioembolic sources, CPAF: covert paroxysmal atrial fibrillation, CA: cancer-associated, AE: arteriogenic emboli, PE: paradoxical embolism, UE: undetermined embolism. Functional dependency was defined as a modified Rankin Scale score 3–5. OR and 95% CI of functional dependency are shown for each potential cause, with reference to CE. The multivariable model 1 included age, sex, baseline National Institutes of Health Stroke Scale score (continuous variable), and reperfusion therapy. The multivariable model 2 included age, sex, baseline National Institutes of Health Stroke Scale score categories (mild 0–4, moderate 5–14, and severe ≥15), pre-stroke modified Rankin scale (0 or 1), and reperfusion therapy. Patients who died during hospitalization or within 3 months were excluded from the analysis. Patients who were lost at 3 months were also excluded from the analysis at 3 months.

© 2018 Kiyuna F et al. *JAMA Network Open*.

eTable 3. Association of Embolic Sources With Poor Functional Outcome in the Sensitivity Analysis

		Age- and sex-adjusted			Model 1			Model 2		
	Event (%)	OR	(95% CI)	P	OR	(95% CI)	P	OR	(95% CI)	P
Discharge										
CE, n = 1582	705 (44.6)	1.00	(reference)		1.00	(reference)		1.00	(reference)	
MCS, n = 135	54 (40.0)	0.92	(0.63–1.34)	0.67	0.97	(0.60–1.57)	0.91	1.00	(0.63–1.59)	0.99
CPAF, n = 33	15 (45.5)	1.12	(0.54–2.30)	0.76	0.98	(0.38–2.53)	0.97	1.00	(0.40–2.51)	1.00
CA, n = 54	34 (63.0)	2.46	(1.38–4.39)	0.002	4.12	(2.14–7.95)	<0.001	3.35	(1.67–6.70)	<0.001
AE, n = 418	58 (13.9)	0.22	(0.16–0.30)	<0.001	0.61	(0.43–0.85)	0.004	0.52	(0.37–0.74)	<0.001
PE, n = 173	13 (7.5)	0.15	(0.08–0.27)	<0.001	0.33	(0.17–0.66)	0.002	0.33	(0.17–0.63)	<0.001
UE, n = 859	210 (24.4)	0.51	(0.42–0.62)	<0.001	1.01	(0.79–1.29)	0.94	0.96	(0.75–1.21)	0.71
3 months										
CE, n = 1562	635 (40.7)	1.00	(reference)		1.00	(reference)		1.00	(reference)	
MCS, n = 134	45 (33.6)	0.83	(0.56–1.23)	0.35	0.80	(0.48–1.33)	0.39	0.86	(0.53–1.40)	0.55
CPAF, n = 33	10 (30.3)	0.66	(0.30–1.46)	0.31	0.39	(0.14–1.11)	0.08	0.44	(0.17–1.15)	0.09
CA, n = 54	40 (74.1)	5.50	(2.89–10.48)	<0.001	10.87	(5.38–21.99)	<0.001	9.25	(4.45–19.23)	<0.001
AE, n = 415	60 (14.5)	0.28	(0.20–0.37)	<0.001	0.81	(0.57–1.14)	0.22	0.72	(0.51–1.02)	0.07
PE, n = 170	9 (5.3)	0.13	(0.06–0.26)	<0.001	0.29	(0.13–0.65)	0.002	0.28	(0.13–0.61)	0.001
UE, n = 846	169 (20.0)	0.48	(0.39–0.59)	<0.001	0.94	(0.72–1.21)	0.61	0.90	(0.70–1.15)	0.40

OR: odds ratio, CI: confidence interval, CE: cardioembolic stroke, MCS: minor-risk potential cardioembolic sources, CPAF: covert paroxysmal atrial fibrillation, CA: cancer-associated, AE: arteriogenic emboli, PE: paradoxical embolism, UE: undetermined embolism. Poor functional outcome was defined as a modified Rankin Scale score 3–6. OR and 95% CI of functional dependency are shown for each potential cause, with reference to CE. The multivariable model 1 included age, sex, baseline National Institutes of Health Stroke Scale score (continuous variable), and reperfusion therapy. The multivariable model 2 included age, sex, baseline National Institutes of Health Stroke Scale score categories (mild 0–4, moderate 5–14, and severe ≥15), pre-stroke modified Rankin scale (0 or 1), and reperfusion therapy. Patients who were lost at 3 months were excluded from the analysis at 3 months.

© 2018 Kiyuna F et al. *JAMA Network Open.*

eTable 4. Adverse Events During Hospitalization and at 3 Months

				ESUS						
	CE	ESUS		MCS	CPAF	CA	AE	PE	UE	
	(n = 2261)	(n = 2163)	P	(n = 209)	(n = 43)	(n = 79)	(n = 522)	(n = 190)	(n = 1120)	P
In-hospital events										
Stroke recurrence	126/2261 (5.6)	106/2163 (4.9)	0.32	11/209 (5.3)	1/43 (2.3)	16/79 (20.3) [a]	20/522 (3.8)	4/190 (2.1)	54/1120 (4.8)	<0.001
Ischemic stroke	114/2261 (5.0)	93/2163 (4.3)	0.24	11/209 (5.3)	1/43 (2.3)	14/79 (17.7) [a]	19/522 (3.6)	3/190 (1.6)	45/1120 (4.0)	<0.001
Hemorrhagic stroke	14/2261 (0.6)	15/2163 (0.7)	0.76	0/209 (0.0)	0/43 (0/0)	2/79 (2.5)	1/522 (0.2)	1/190 (0.5)	11/1120 (1.0)	0.12
Mortality	127/2261 (5.6)	40/2163 (1.8)	<0.001	4/209 (1.9) [a]	0/43 (0.0)	14/79 (17.7) [a]	1/522 (0.2) [a]	1/190 (0.5) [a]	20/1120 (1.8) [a]	<0.001
3-month events										
Stroke recurrence	162/2225 (7.3)	162/2135 (7.6)	0.70	14/206 (6.8)	2/43 (4.7)	22/76 (28.9) [a]	36/517 (7.0)	7/187 (3.7)	81/1106 (7.3)	<0.001
Ischemic stroke	145/2225 (6.5)	145/2133 (6.8)	0.71	13/206 (6.3)	2/43 (4.7)	20/76 (26.3) [a]	35/517 (6.8)	4/187 (2.1)	71/1104 (6.4)	<0.001
Hemorrhagic stroke	19/2222 (0.9)	21/2133 (1.0)	0.65	1/206 (0.5)	0/43 (0.0)	2/75 (2.7)	1/517 (0.2)	3/187 (1.6)	14/1105 (1.3)	0.09
Mortality	209/2230 (9.4)	98/2135 (4.6)	<0.001	14/206 (6.8)	1/43 (2.3)	37/77 (48.1) [a]	5/517 (1.0) [a]	2/187 (1.1) [a]	39/1105 (3.5) [a]	<0.001

ESUS: embolic stroke of undetermined source, CE: cardioembolic stroke, MCS: minor-risk potential cardioembolic sources, CPAF: covert paroxysmal atrial fibrillation, CA: cancer-associated, AE: arteriogenic emboli, PE: paradoxical embolism, UE: undetermined embolism. Data are expressed as number of events/numbers of patients (%). Patients with missing data on stroke recurrence or mortality at 3 months were excluded from the analysis for adverse events at 3 months. [a] P <0.05 vs. CE by multiple comparison test.

© 2018 Kiyuna F et al. *JAMA Network Open.*

eTable 5. Background Characteristics of Patients With Complete and Incomplete Data on 3-Month Outcome

	Incomplete	Complete	
	(n = 28)	(n = 2135)	P
Age, mean (SD), y	73.1 (14.5)	72.4 (12.6)	0.61
Males, n (%)	17 (60.7)	1218 (57.0)	0.70
Risk factors, n (%)			
Hypertension	22 (78.6)	1625 (76.1)	0.76
Diabetes mellitus	9 (32.1)	627 (29.4)	0.83
Dyslipidemia	11 (39.3)	1101 (51.6)	0.20
Smoking	17 (60.7)	1111 (52.0)	0.36
Drinking	10 (35.7)	663 (31.1)	0.68
Comorbidity, n (%)			
Chronic kidney disease	13 (46.4)	944 (44.2)	0.81
Coronary artery disease	4 (14.3)	339 (15.9)	1.00
Pre-stroke dependency, n (%)	8 (28.6)	483 (22.6)	0.50
NIHSS score on admission, median (IQR)	2.5 (1–8.75)	3 (1–6)	0.87
Reperfusion therapy, n (%)	4 (14.3)	198 (9.3)	0.33
Thrombolytic therapy	4 (14.3)	186 (8.7)	0.30
Endovascular thrombectomy	1 (3.6)	30 (1.4)	0.33
Antithrombotic therapy, n (%) [a]	(n = 28)	(n = 2095)	
Antiplatelets	15 (53.6)	1460 (69.7)	0.07
Anticoagulants	13 (46.4)	656 (31.3)	0.10

NIHSS: National Institutes of Health Stroke Scale, IQR: interquartile range. Background characteristics were compared between patients included and excluded because of lack of data on functional outcome at 3 months. [a] Data are expressed as numbers of patients (%) after excluding patients who died during hospitalization.

© 2018 Kiyuna F et al. *JAMA Network Open.*
